# Pre & Postsynaptic Tuning of Action Potential Timing by Spontaneous GABAergic Activity

**DOI:** 10.1371/journal.pone.0022322

**Published:** 2011-07-15

**Authors:** Olivier Caillard

**Affiliations:** 1 INSERM UMR641, IFR Jean-Roche, Marseille, France; 2 Université de la Méditerranée, Marseille, France; Centre national de la recherche scientifique, University of Bordeaux, France

## Abstract

Frequency and timing of action potential discharge are key elements for coding and transfer of information between neurons. The nature and location of the synaptic contacts, the biophysical parameters of the receptor-operated channels and their kinetics of activation are major determinants of the firing behaviour of each individual neuron. Ultimately the intrinsic excitability of each neuron determines the input-output function. Here we evaluate the influence of spontaneous GABAergic synaptic activity on the timing of action potentials in Layer 2/3 pyramidal neurones in acute brain slices from the somatosensory cortex of young rats. Somatic dynamic current injection to mimic synaptic input events was employed, together with a simple computational model that reproduce subthreshold membrane properties. Besides the well-documented control of neuronal excitability, spontaneous background GABAergic activity has a major detrimental effect on spike timing. In fact, GABA_A_ receptors tune the relationship between the excitability and fidelity of pyramidal neurons via a postsynaptic (the reversal potential for GABA_A_ activity) and a presynaptic (the frequency of spontaneous activity) mechanism. GABAergic activity can decrease or increase the excitability of pyramidal neurones, depending on the difference between the reversal potential for GABA_A_ receptors and the threshold for action potential. In contrast, spike time jitter can only be increased proportionally to the difference between these two membrane potentials. Changes in excitability by background GABAergic activity can therefore only be associated with deterioration of the reliability of spike timing.

## Introduction

Variability in neural activity is apparent throughout the central nervous system, in a wide range of electrophysiological signals. Several well recognized sources of noise in cortical neurons contribute to spike train variability, like the probabilistic release of transmitter and the stochastic gating of ion channels [1], [2]. Evoked activities in stimuli-driven experiments are always superimposed on ongoing background activity, fluctuations of which contribute to the *in vivo* trial-to-trial variability [3]–[6]. Ongoing subthreshold activity of neocortical neurones is characterised by alternating states of low or intense synaptic activity [7], [8], supported at least in part by two major neurotransmitters, GABA and glutamate. GABA activates mainly GABA_A_ receptors, which first reduce the excitability of the cells by reducing the membrane resistance and second, according to their reversal potential, will bring the membrane potential away or close to the threshold for action potentials [9]–[11].

Two main functional roles have been established for spontaneous GABAergic activity. The first relates to the membrane time constant of the cell: if the passive properties of the cell are comparable to a resistor-capacitor circuit, with a capacitance proportional to the surface of plasma membrane, and a resistance inversely proportional to the number of open ion channels, strong activation of GABA_A_ receptors leads to a decrease in the membrane time constant, which in turn speeds up dV/dt. As a consequence, individual excitatory postsynaptic currents are better resolved in time, and voltage changes are steeper [8], [12], [13]. The second is attributed to the background noise that creates a dynamic range in the input/output function of a neuron: referred to as stochastic resonance [14], [15]: the processing of synaptic events by background activity, according to noise level or variability, translates the information provided by excitatory events into firing probability, despite the fact that this process is nonlinear [15]–[17]. Nevertheless, compelling evidence supports the fact that, even in individual cells, not only the average frequency of firing but also the timing of each action potential is relevant for neuronal information coding. Reliability in spike timing is known to be dependent on the structure of the excitatory stimulus [18]. The sequential activation of excitatory and inhibitory inputs in feed-forward networks [19] and the presence of functional GABAergic autapses at some fast-spiking interneurones [20] were shown to be relevant physiological mechanisms that can reinforce spike timing in neuronal networks. In spite of that, since most of the synaptic activity received either *in vitro* or *in vivo* appears to be uncorrelated to any specific stimulus, a yet unsolved question is what influence spontaneous GABAergic activity has on the temporal fidelity of pyramidal cell firing, according to the mode of activation of GABA_A_ receptors [21] or their reversal potential [22].

Here we evaluate and quantify how spontaneous activation of GABA_A_ receptors affects action potential firing and timing, according to the frequency of action potential discharge, and various modes of GABA release. We also evaluate the relative contribution of shunting inhibition and driving force for GABA_A_ receptors to neuronal discharge.

## Results

### Spontaneous GABAergic activity tunes the temporal fidelity of Pyramidal Cell discharge

**Figure 1:**
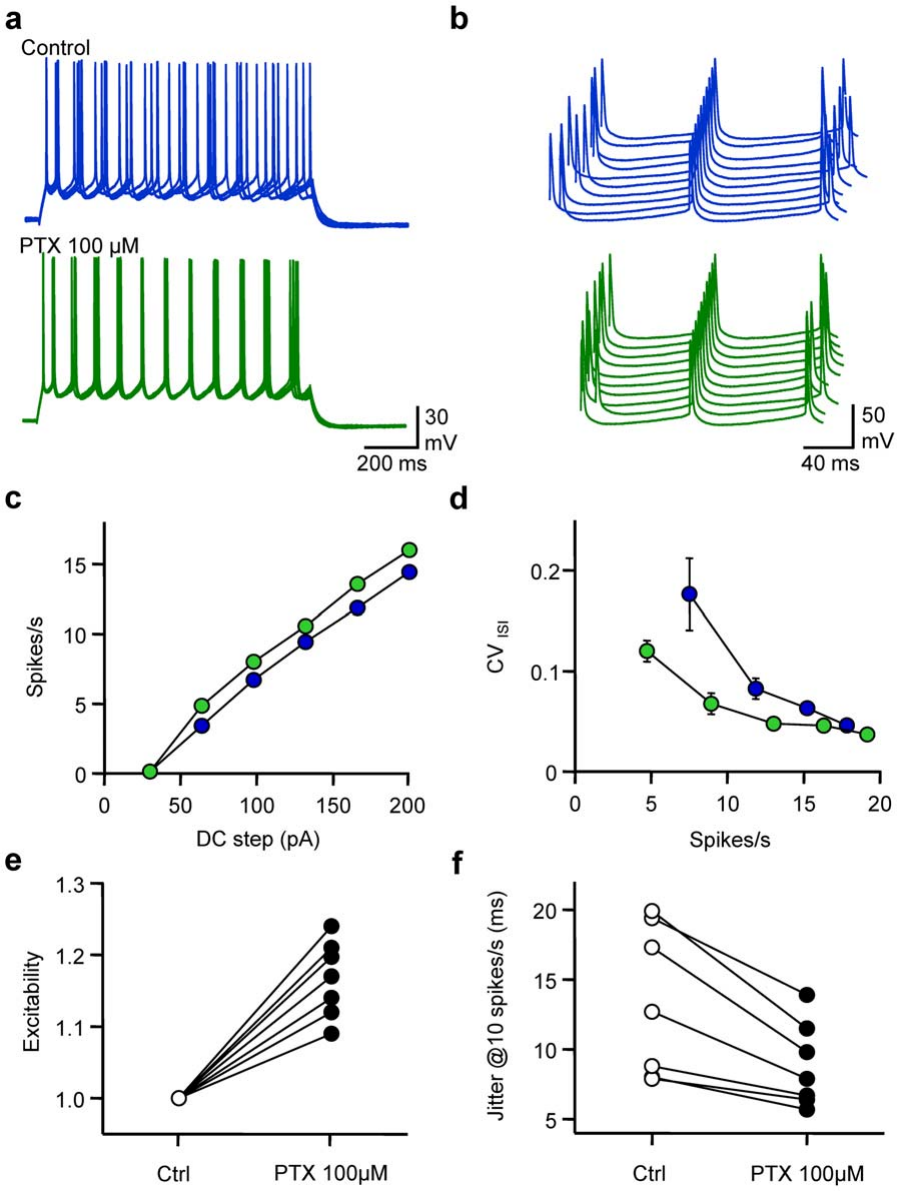
Spontaneous GABAergic activity affects excitability and spike timing of pyramidal cells. a, Superimposed (5) membrane potential (Vm) fluctuations of a gramicidin-perforated current-clamped L2/3 pyramidal cell in response to a DC step (1 s, 170 pA) before (control, blue) and after extracellular application of picrotoxin (100 µM, PTX, green). The cell was held at around −80 mV in both conditions. Same cell and colour code for b, c and d. b, Waterfall view of Vm when the 5^th^ spike was set as the time reference. c, Mean firing rate *vs* DC step. d, Coefficient of Variation of the Inter Spike Interval (CV_ISI_) *vs* firing rate. e, Normalised changes in excitability observed when adding PTX (n = 7). f, Changes in spike jitter for an interpolated firing rate of 10 spikes/s.

In order to evaluate the impact of spontaneous GABAergic activity received by L2/3 pyramidal neurones on their spike timing, gramicidin-perforated patch-clamp recordings Fig. 1). Although several precautions were taken, the possibility of an intermediated perforated-patch/whole-cell configuration cannot be completely excluded (see methods). Excitability, defined as the average firing rate, and fidelity measured as the average coefficient of variation of inter-spike intervals along the discharge (CV_ISI_, see methods) were calculated for various DC steps. The influence of GABAergic activity on these parameters was tested by application of picrotoxin (PTX, 100 µM) a GABA_A_ receptor blocker. Blockage of GABA_A_ receptors, whilst not affecting resting Vm (average change −0.2±0.9 mV, n = 7, P = 0.86) increased the excitability, reflected by a leftward shift of the frequency *vs* current relationship of recorded cells. Although CV_ISI_ was dependent on firing frequency in both control conditions and when GABA_A_ receptors were blocked, the CV_ISI_ was reduced at comparable firing rates in the presence of PTX, especially for low rates of AP firing. When changes in excitability were normalised for each individual cell as the average change in spike number at each DC step, a significant increase in intrinsic excitability was observed (117±5%, n = 7, P<0.001). Spike time jitter, defined as the standard deviation of the average ISI, was calculated when interpolating CV_ISI_ at a firing rate of 10 spikes/s. While spike time jitter was quite heterogeneous from one cell to another in control conditions (range 6.6–19.9 ms), it was always reduced after PTX application. On average, blocking GABA_A_ receptors significantly reduced spike time jitter from 13.4±5.4 ms to 8.8±3.0 ms (68±10%, n = 7, P<0.05). Thus, GABAergic activity controls both excitability and fidelity of pyramidal cell discharge.

### Presynaptic control of temporal fidelity

**Figure 2:**
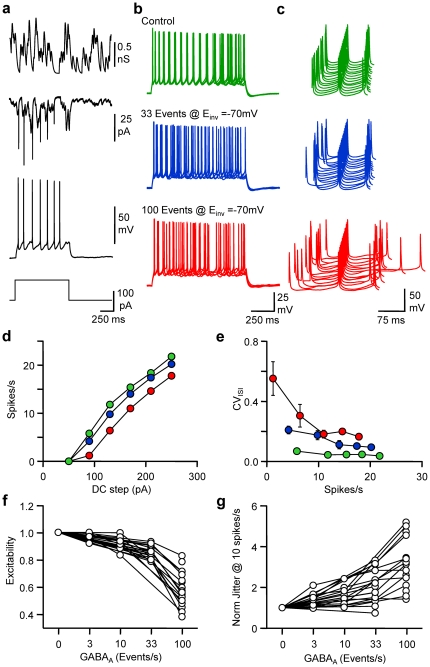
Frequency-dependent tuning of pyramidal cell excitability and spike timing. a, From upper to lower, an example of a GABA_A_ conductance (G_GABA-A_) pattern of 33 events/s, GABA_A_ current (I_GABA-A_), Vm and the DC protocol in a dynamic clamp recording of a L2/3 pyramidal cell. b, Superimposed (5) Vm fluctuations in response to a DC step (1 s, 170 pA) in control (green), in the presence of 33 (blue) or 100 (red) randomly occurring GABA_A_ events/s dynamically injected with E_GABA_ set at −70 mV. Same cell and colour code for c,d and e. c, Waterfall view of Vm when the 5^th^ spike was set as the time reference. d, Mean Firing rate *vs* DC step. e, CV_ISI_ *vs* mean spike firing rate. f, Normalised changes in excitability observed when neurons receive different levels of randomly occurring GABA_A_ events/s. g, Normalised changes in spike jitter for an interpolated firing rate of 10 spikes/s.

In order to quantify the contribution of various levels of GABAergic activity to pyramidal cell discharge parameters, currents mimicking spontaneous GABAergic events were injected in whole-cell recorded neurones by means of a dynamic-clamp amplifier in the presence of PTX (100 µM). Patterns of spontaneous GABAergic synaptic conductances were constructed by convoluting the occurrence in time of unitary events with a conductance transient template that had similar characteristics to the average event measured experimentally (Fig. S1). The time of occurrence of each event obeyed a Poisson law and was determined extemporaneously according to the desired target rate of spontaneous activity. In a first set of experiments the reversal potential for GABA_A_ receptors (E_GABA_) was set at −70 mV (Fig. 2). Experiments were performed at rates of GABAergic events going from 0 (control conditions) to 100 events/s. Both excitability and fidelity varied according to the level of GABA_A_ activity. When changes in excitability were normalised for each recorded neuron, 3-10-33-100 events/s reduced excitability by 0.3±1% (n = 7, P = 0.085), 8±2% (n = 9, P<0.001), 15±1% (n = 14, P<0.0001) and 44±3% (n = 12, P<0.0001) respectively. When spike time jitter at a firing rate of 10 spike/s was normalised in the absence of GABAergic activity for each recorded cell, 3-10-33-100 events/s increased spike time jitter by 148±14% (n = 7, P<0.05), 157±15% (n = 9, P<0.005), 209±20% (n = 17, P<0.0001), 316±32% (n = 15, P<0.0001) respectively. Thus, spontaneous GABA_A_ activity not only reduces neuronal excitability, but has a far more significant effect in deteriorating spike time precision.

### Fidelity, not excitability depends on the mode of GABA_A_ activation

**Figure 3:**
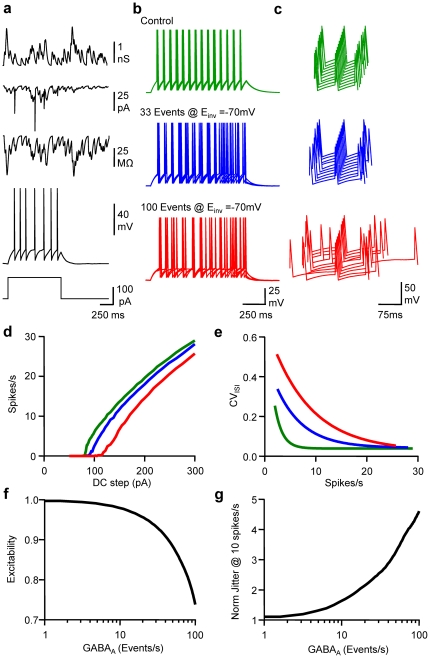
Modelling frequency-dependent tuning of neuronal excitability and spike timing. a, From upper to lower, an example of G_GABA-A_ pattern of 33 events/s, I_GABA-A_, R_in_, Vm and a DC step (1 s, 120 pA) when running the LIF model. b, Superimposed (5) Vm fluctuations in response to a DC step (1 s, 160 pA) in control (green), in the presence of 33 (blue) or 100 (red) randomly occurring GABA_A_ events/s with E_GABA_ set at −70 mV. Same cell and colour code for c,d and e. c, Waterfall view of Vm when the 5^th^ spike was set as time reference. d, Mean Firing rate *vs* DC step. e, CV_ISI_ *vs* firing rate. f, Normalised changes in excitability observed when LIF received 1 to 100 randomly occurring GABA_A_ events/s. g, Normalised changes in spike jitter for a firing rate of 10 spikes/s.

In order to verify the conclusions drawn from the experimental data and further analyse the correlation between the rate of the spontaneous activity input, the output firing rate and the fidelity of neuronal discharge, we used a “leaky integrate and fire” with Random action potential Threshold (RT-LIF) model [23], [24], in order to focus upon the subthreshold membrane properties according to GABA_A_ activation while excluding the mechanisms responsible for action potential triggering. The standard deviation of the threshold was set to 1 mV so that frequency-dependent jittering resembling the experimental data was observed. The model consistently reproduced the firing behaviour of L2/3 pyramidal cells with realistic membrane time constant in the absence of synaptic activity (Fig. S2). Both excitability and fidelity were affected proportionally to the level of GABA_A_ activity (Fig. 3). As for dynamic-clamp *in vitro* experiments, spike time jitter evaluated from RT-LIF was much more prone to changes, according to GABA_A_ activity and firing rate. Thus for a rate of 10 spikes/s, 8 GABA_A_ events/s were sufficient to increase jitter by 50%, while reducing excitability by only 2%. Both the transient changes in current and membrane resistance associated with GABA_A_ activity contributed to these effects (Fig. S3) and when one or the other constituent was annulled, the impact of changes in GABA_A_ activity on excitability and fidelity was reduced. The charge of the random GABA_A_ conductance transients determined the impact of random GABA_A_ activity on both excitability and spike timing. Indeed, the detrimental effects of GABA_A_ activity were proportional to the peak GABA_A_ conductance (Fig. S4), while for a fixed GABA_A_ charge, slowing or speeding up GABA_A_ conductance transient kinetics did not affect the impact of GABA_A_ activity on neither excitability or spike timing (Fig. S5).

The influence of the waveform pattern of GABA_A_ activity was then tested on the number and precision of spikes emitted during the injection of various DC steps: the first mimicked the experimental spontaneous activity received by pyramidal neurones *in vitro* (Fig. S1); the second mode corresponded to an invariable pattern of GABA_A_ conductance transients but consistently replayed, so that these G_GABA-A_ transients were time-locked during the different simulations; the third mode corresponded to a constant G_GABA-A_, i.e. tonic activity [21], which was an average over time of the conductance transients received by the RT-LIF in the previous two modes.

**Figure 4:**
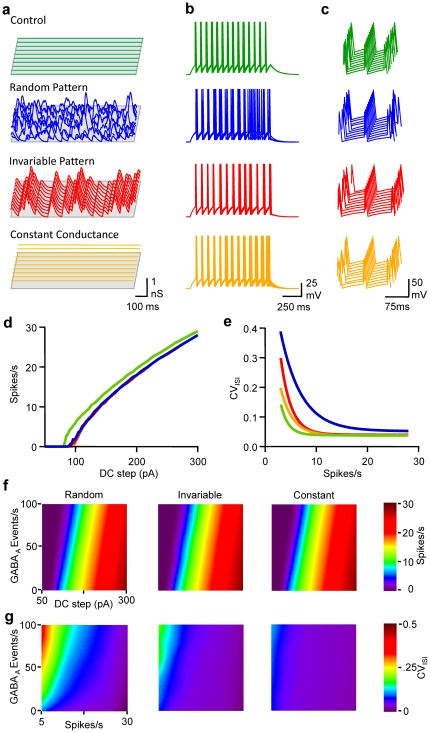
Randomly occurring GABA_A_ conductance transients underlie the frequency-dependent tuning of pyramidal cell discharge fidelity. a, Waterfall view of conductance fluctuations injected into the LIF model in control conditions (no conductance, green), when the LIF model received 33 randomly occurring GABA_A_ events/s (blue), an invariable pattern of 33 GABA_A_ events/s (red), or when the neuron model received a constant G_GABA-A_ equivalent to the average conductance for a rate of 33 events/s (orange). Same cell and colour code for b,c,d and e. b, Superimposed (5) Vm fluctuations of the LIF model in response to a DC step (1 s, 160 pA) in the various conditions depicted in a. c, Waterfall view of Vm when the 5^th^ spike was set as the time reference. d, Mean firing rate *vs* DC step. e CV_ISI_ *vs* firing rate. f, Mean firing rate displayed on a pseudocolor scale *vs* DC step and rate of GABA_A_ activity when LIF model received random (left), invariable (middle) or constant conductance (right). g, CV_ISI_ displayed on a pseudocolor scale *vs* firing and GABA_A_ activity rate. Same conditions as in f.

When compared to control conditions, i.e. no GABAergic activity, a rate of 33 randomly occurring G_GABA-A_ transients per second notably increased spike time jitter, and both excitability and the fidelity of discharge were reduced (Fig. 4). When GABA_A_ conductance transients were evoked invariably in time, despite the fact that the excitability was modified to the same extent as with random input, spike time jitter was much less affected. Although CV_ISI_ was larger than in control conditions, especially at low firing rates, it was smaller than the conditions in which randomly occurring spontaneous activity was generated. Last, the impact of a constant activation of GABA_A_ conductance was tested (i.e. tonic activation of GABA_A_ receptors [21]). Again, the decrement in excitability was comparable to other modes of GABA_A_ activation, but CV_ISI_ *vs* firing rate relationship was close to control conditions. In order to get a comprehensive view of the impact of the different modes of GABA_A_ activation, FI curves were drawn for a range of 0 to 100 events/s in the 3 modes defined above. While FI curves were comparably shifted to the right by GABA_A_ activity in a frequency dependent manner, whatever the mode, the effect of GABA_A_ activity on CV_ISI_ was highly dependent on the mode. In fact, spike-timing fidelity was noticeably reduced only if the cell, firing at low rate, received a high level of random GABA_A_ activity.

**Figure 5:**
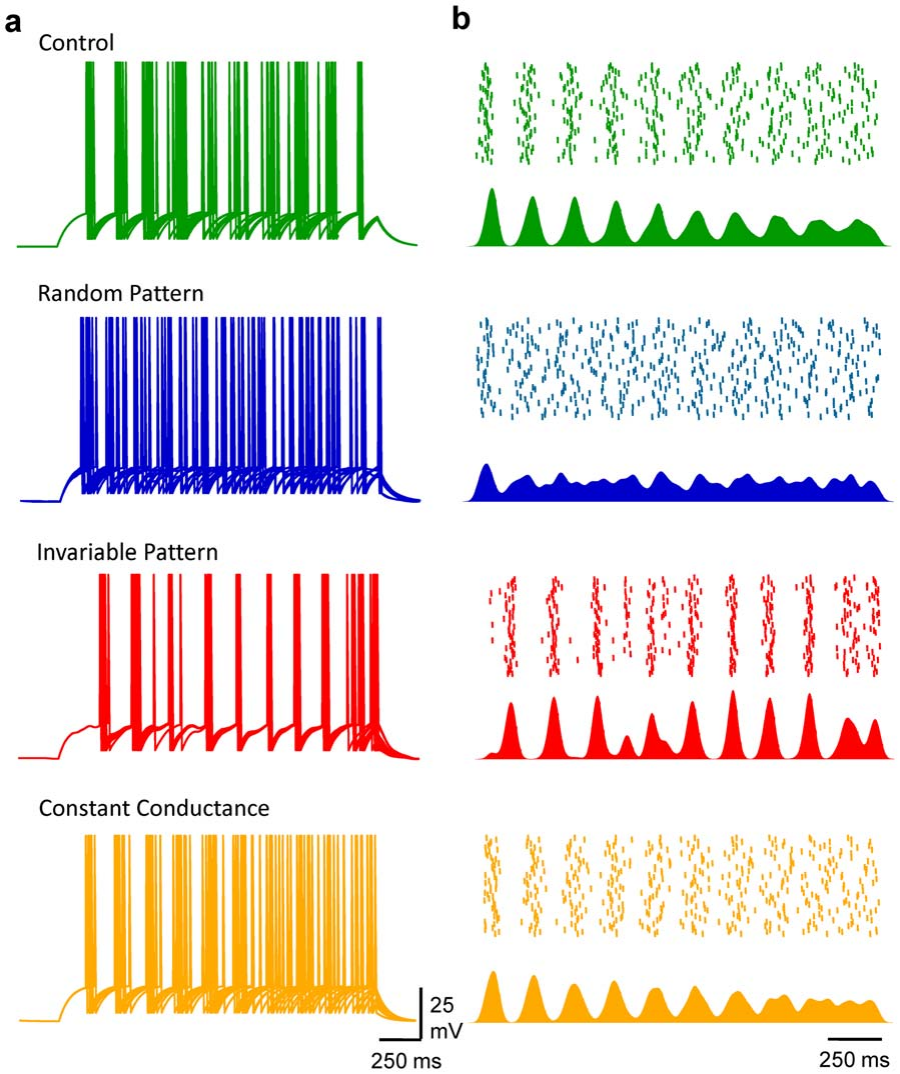
Randomly occurring GABA_A_ conductance transients underlie the frequency-dependent tuning of pyramidal cell discharge fidelity. a, Superimposed (10) Vm fluctuations of the LIF model in response to a DC step (2 s) that allows a firing rate of 5 spikes/s, in control conditions (green, 95 pA), when the LIF model received 33 randomly occurring GABA_A_ events/s (blue, 111 pA), an invariable pattern of 33 GABA_A_ events/s (red, 110 pA), or when the neuron model received a constant G_GABA-A_ equivalent to the average conductance for a rate of 33 events/s (orange, 110 pA). b up, Raster plot of spike times collected from 50 consecutive trials in the various conditions depicted in a; down, Spike probability over time for these 50 consecutive trials.

In order to confirm that GABA_A_ activation modes can affect spike timing in a different ways, an alternative type of spike timing analysis was performed (Fig. 5). Thus, instead of analysing the variability of the ISI along the discharge, the timing of action potentials initiated over 50 presentations was measured in control conditions and in the 3 different modes of GABA_A_ activation. The DC step was adjusted in order to get an average rate of 5 spikes/s in all 4 conditions. Once each AP was detected, the timing data was convolved with a Gaussian waveform, in order to reflect both reliability and temporal precision of spike initiation. In control conditions, temporal organisation faded with time, but was still relatively high at the end of the discharge. In contrast, random patterns (33 events/s) of GABAergic activity rapidly disrupted the temporal organisation of the discharge. When the pattern of GABAergic activity was invariably replayed, peaks of AP initiation probability were higher in number than in control conditions, and varied in amplitude and width such that for specific time intervals AP initiation probability was higher than in control conditions. Last, in the presence of tonic activity, temporal dispersion and variance waveform were close to control conditions until the last third of the discharge.

Thus, in contrast to invariable phasic or tonic GABA_A_ activity, randomly occurring spontaneous GABA_A_ activity has a major detrimental effect on spike timing.

### Postsynaptic control of spike timing fidelity

**Figure 6:**
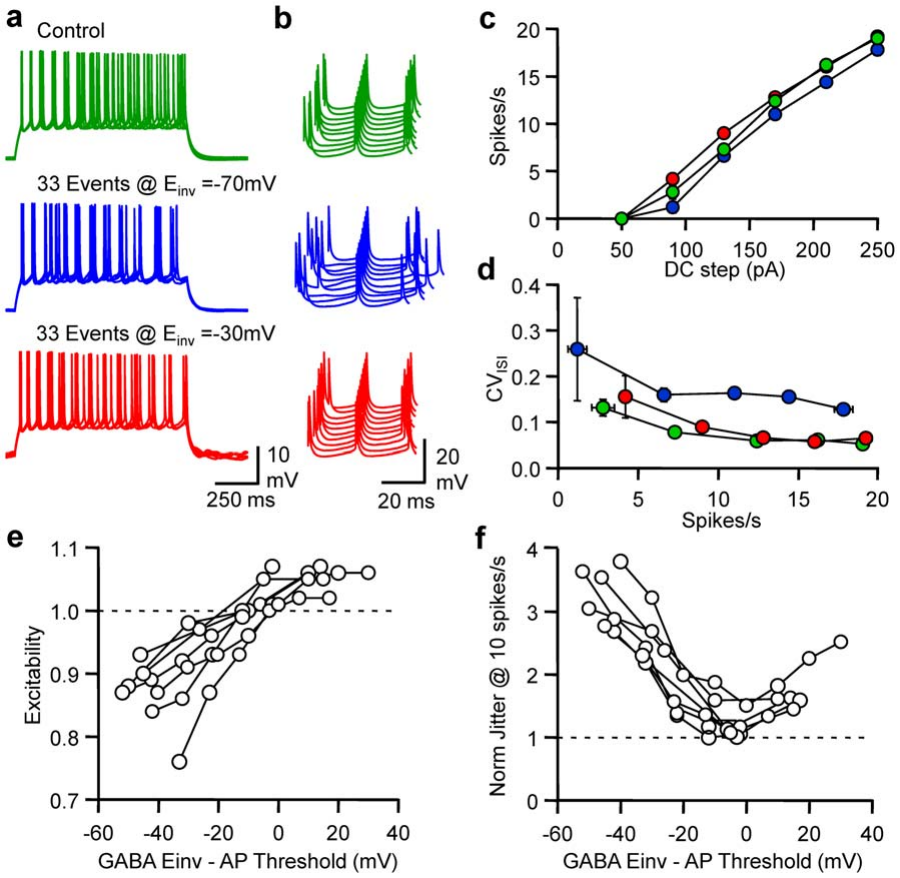
Reversal potential-dependent tuning of pyramidal cell excitability and discharge fidelity. a, Superimposed (5) Vm fluctuations of a whole-cell L2/3 pyramidal neurone in response to a DC step (1 s, 170 pA) in control conditions (green), when 33 randomly occurring GABA_A_ events/s were dynamically injected at E_GABA_ = −70 mV (blue) or −30 mV (red). Same cell and colour code for b, c and d. b, Waterfall view of Vm when the 5^th^ spike was set as the time reference. c, Mean firing rate *vs* DC step. d, CV_ISI_ *vs* firing rate. e, Normalised changes in excitability observed when neurons received 33 randomly occurring GABA_A_ dynamic currents/s at various differences between E_GABA_ and the calculated threshold for AP in each individual cell (n = 9). f, Normalised changes in spike jitter observed for a firing rate of 10 spikes/s. Same conditions as in e.

In order to test the influence of E_GABA_ on excitability and fidelity, dynamic currents mimicking spontaneous GABAergic events were injected in pyramidal neurones at an average rate of 33 events/s in the presence of PTX (100 µM). The effects of spontaneous GABA_A_ transients were first compared for E_GABA_ = −70 mV and −30 mV (Fig. 6). While a decrease in excitability and an increase in spike jitter was observed for E_GABA_ = −70 mV, a slight increase in excitability was noted for E_GABA_ = −30 mV, while the spike jitter was similar to control conditions. Quantification of changes in excitability at various firing rates showed that the FI curve was shifted either leftward or rightward according to E_GABA_. While major changes in CV_ISI_ were observed for all firing rates tested at E_GABA_ = −70 mV, they were negligible at E_GABA_ = −30 mV. A series of experiments were performed with a broad range of E_GABA_ (from −80 to 0 mV) in order to establish the relationship between E_GABA_, excitability and fidelity. Pyramidal neurone excitability was linearly dependent on E_GABA_ and changes in excitability were abolished when E_GABA_ was 6.1 mV under the AP threshold (n = 9; 95% confidence interval 10.3/1.9 mV under AP threshold). The relationship between normalised jitter for an interpolated firing rate of 10 spikes/s was bell-shaped, with a minimum of 1.33 (n = 9; 95% confidence interval 1.12/1.54) for E_GABA_ being 2.5 mV under the AP threshold.

### On/Off Control in Spike Timing tuning by GABA_A_ reversal potential

**Figure 7:**
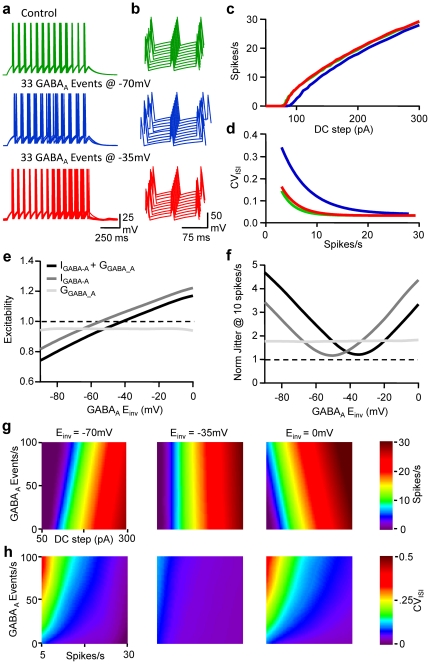
Frequency and reversal potential-dependent tuning of pyramidal cell excitability and discharge fidelity. a, Superimposed (5) Vm fluctuations of the LIF model in response to a DC step (1 s, 160 pA) in control conditions (green), when the LIF model received 33 randomly occurring GABA_A_ events/s at E_GABA_ = −70 mV (blue) or −35 mV (red). Same colour code for b, c and d. b, Waterfall view of Vm when the 5^th^ spike was set as the time reference. c, Mean firing rate *vs* DC step. d, CV_ISI_ *vs* firing rate. e, Normalised changes in excitability observed when LIF model received 33 randomly occurring GABA_A_ events/s at various E_GABA_ in control conditions (black), when GABA activity induced only transient changes in I_GABA-A_ (gray) or when GABA activity induced only transient changes in R_in_ (light gray). f, Normalised changes in spike jitter observed for a rate of 10 spikes/s. Same conditions as in e. g, From left to right firing rate displayed on a pseudocolor scale *vs* DC step and E_GABA_ when the LIF model received 33 randomly occurring GABA_A_ events/s in control conditions (left), when GABA_A_ activity induced only transient changes in I_GABA-A_ (middle) or when GABA activity induced only transient changes in R_in_ (right). h, CV_ISI_ displayed on a pseudocolor scale *vs* firing rate and E_GABA_. Same conditions as in g.

To further analyze the relationship between the reversal potential for GABA_A_ receptors, the rate of firing and spike timing, an initial simulation was performed with RT-LIF receiving an input rate of 33 random GABA_A_ conductance transients per second. E_GABA_ and DC steps varied from −90 to 0 mV and 50 to 300 pA, respectively (Fig. 7). While an increase in spike jitter, associated with an increase in interspike interval was obvious for E_GABA_ = −70 mV, it was similar to control conditions for E_GABA_ = −35 mV. Both transient changes in current and membrane resistance led to changes in excitability and fidelity (Fig. 7). Modifications in excitability were abolished when E_GABA_ was 5 mV above AP threshold. When considering spike timing, a bell-shaped relationship between CV_ISI_ and E_GABA_ was observed with a minimum at 10 mV above AP threshold. When transient changes in membrane resistance were annulled, changes in excitability were abolished if E_GABA_ was 10 mV under AP threshold and the CV_ISI_ was minimal when E_GABA_ was 5 mV under AP threshold. Thus GABA_A_ currents can almost compensate for the decrease in the fidelity of spike timing and excitability induced by the reduction in membrane resistance when E_GABA_ is slightly above the AP threshold.

In order to get a better view of the impact of spontaneous GABA_A_ activity on excitability and spike time jitter at different E_GABA_, FI curves were drawn for a range of 0 to 100 events/s at 3 different E_GABA_. FI curves were shifted right and left by GABA_A_ activity in a frequency dependent manner for E_GABA_ = −70 mV and E_GABA_ = 0 mV respectively. However at E_GABA_ = −35 mV increasing GABA_A_ activity had no influence on the relationship between DC step and average firing rate. Analysis of the influence of random GABA_A_ activity on CV_ISI_ at these 3 different E_GABA_ indicated that while the level of GABA_A_ activity had a major influence on CV_ISI_ at E_GABA_ = −70 or 0 mV, it had little effect on CV_ISI_ at E_GABA_ = −35 mV.

Thus, by regulating their GABA_A_ receptor reversal potential, i.e. their intracellular chloride concentration [22], [25], neurones retain control of how spontaneous GABAergic activity may increases or decrease excitability but also to which level the temporal fidelity of their discharge will be deteriorated.

## Discussion

Ongoing background activity has been shown to have an important impact on neuronal dynamics. Changes in the level of background inhibition alter the electrical compactness and modify the integrative properties [13], [26], modulate the gain [27] and thus can dynamically set the range of the input/output function of neurones [15]–[17]. Here I have emphasized the fact that GABAergic activity not only affects the excitability of pyramidal cells, but also alters spike timing in proportions that can be significantly higher, according to its temporal organisation and E_GABA_.

### Variations in background GABAergic activity

Ongoing subthreshold activity of neocortical neurones is subject to important changes. In the anaesthetised animal, alternating states of low or intense activity can be observed: the down-states during which little or no synaptic background activity is visible and the up-states when the cells are continuously bombarded by spontaneous activity [7], [8], [26]. The pattern of GABAergic activity that is received by these cells is determined by the number, the variety and activity of presynaptic interneurones [28]. The complexity of the GABAergic system is enhanced by the fact that GABAergic synapses, according to their location, exhibit specific electrophysiological features [29]–[32]. Additionally, the level of spontaneous GABAergic activity is subject to prominent changes during development [33], short [34], [35] or long-term plasticity [36]–[43] and can be altered in different pathologies [44]–[47].

In the absence of synaptic activity, the spike generating mechanism shows a certain level of reliability and precision [18] that can be noticeably reduced by the spontaneous occurrence of few GABA_A_ synaptic events. Experiments performed *in vitro* with endogenous GABAergic activity or computer-modelled background activity injected to the cell by the means of a dynamic-clamp amplifier could be very well predicted with a RT-LIF model. Therefore, while taking into account the fact that the experiments presented here represent only a very limited range of presynaptic activities when compared to what has been reported *in vivo* during up-states [8], the RT-LIF model appears to be, for this range level of activity, sufficiently complex to capture the essential features of the impact of GABA_A_ activity on excitability and spike-timing.

Beyond their molecular heterogeneity, GABA_A_ receptors can be activated following two different modes, phasic or tonic release. Phasic activation of GABA_A_ receptors relies on synchronous opening of clustered synaptic receptors, while tonic activation results from the activation of extra synaptic receptors [21]. Results obtained in this study show that tonic and phasic inhibition modify to the same extent the input/output relationship, when a repetitive discharge was induced by step excitation. Thus, the average input/output relationship appears to be dependent neither on the timing nor the shape of the spontaneous conductance transients but rather on the average conductance over time (Fig. 4). In contrast, major differences between the mode of activation of GABA_A_ receptors and the fidelity of spike timing were observed. Indeed the increase in variability was dependent on the average GABA_A_ conductance; it appeared first that transient changes in inhibitory conductance were more prone to disorganize firing, and second that random trains were more potent than fixed patterns of experimentally derived synaptic conductance waveforms.

In fact, from a control condition where the action potential delay is dependent on the AHP amplitude, the membrane time constant and the voltage threshold for action potential, transient inhibitory conductances delay Vm from reaching action potential threshold and thus induce both an increase in ISI mean and variability, such that the temporal structure of neuronal discharge is rapidly lost. If now the timing of transient inhibitory conductances remains invariable, the probability of firing an action potential is not uniform but varies according to the pattern of inhibitory conductance (Fig. 5). As demonstrated by Mainen and Sejnowski [18], when a DC pattern is invariably replayed, the reliability of the firing pattern increases as a function of the fluctuation amplitude. Tiesinga and Toups [48] later showed that an invariable fluctuating DC pattern uncovers, after a statistical sorting of the different trials, a series of precise spike patterns with different spike times and eventually a different number of spikes [49]. Thus, the variance of inputs uncovers different spike patterns and maintains a high probability of spike timing in narrow windows all along the discharge, but increases the variability of the ISI. In contrast tonic inhibition, whatever the analysis method employed (CV_ISI_ or firing probability over time) barely deteriorates spike timing. Pouille and Scanziani [19] have demonstrated that feed forward inhibition curtailed EPSPs and improved EPSP-spike timing *in vitro*. In some specific GABAergic interneurones, because autaptic transmission offers a tight temporal link between the spike and GABAergic conductance, the reliability of spike timing is greatly improved by the activation of autaptic GABA_A_ receptors [20]. Despite the fact that in these two latter examples, where a subpopulation of GABAergic synapses is activated sequentially after some excitatory synaptic inputs or neuronal discharge, the temporal structure of most of the GABAergic activity is often uncorrelated to pre or postsynaptic activity. From the results described here, reducing the fidelity of discharge by adding tonic or phasic inhibition may at first sight appear contradictory with previously published results. However one has to take into account the fact that here GABA_A_ activity was not time-locked to patterns of EPSPs and also that fidelity was measured and compared for similar rates of spike discharge. It demonstrates that, once neurones trigger action potentials at a sustained rate, the consequences of a given amount of GABA, whatever its mode of release i.e. phasic random *vs* phasic invariable vs tonic, is quantitatively the same on excitability, but appears to be different on spike timing. While tonic activation of GABA_A_ receptors contributes to the modulation of excitability and thus may have a key role in epileptogenicity [50], its ability to disorganise firing discharge seems limited. In conclusion, excitability and spike timing modulation by GABA_A_ receptors are not strictly related, but rely on the mode of GABA release and the eventual temporal correlation between the activation of GABA_A_ receptors, the excitatory inputs and action potential firing in case of repetitive discharge.

### Variations in GABA_A_ reversal potential

GABA_A_ receptors are permeable to chloride and bicarbonate ions [51]. As bicarbonate ions are much less permeant than chloride, and because the chloride equilibrium potential is usually more negative than the resting membrane potential, activation of GABA_A_ receptors typically results in a hyperpolarising inhibitory postsynaptic potential. Nevertheless, the chloride gradient is developmentally regulated and subject to plasticity [25]. A large range of GABA_A_ reversal potentials has been reported in the past twenty years [52]. Evoked GABAergic activity was shown to affect the timing of spikes evoked by membrane potential oscillations, according to the phase at which GABA_A_ receptors were activated and E_GABA_ [53]. According to development, the neural situation, depolarised E_GABA_ may be relevant and required for proper network activity, though consideration has to be given that this will modify the temporal organisation of the postsynaptic discharge and the stability of network oscillations [53].

From the present study it appears that intracellular chloride homeostasis has a very important role in defining the changes in excitability and fidelity induced by a given rate of presynaptic GABA release. Spike timing jitter is closely proportional to the absolute difference between the threshold for action potential and E_GABA_. From this study it appears that, for a given rate of GABAergic activity, both the changes in membrane resistance and the currents associated with GABA_A_ conductance affect excitability and spike timing. Excitability and spike time jitter are proportional to GABA_A_ conductance. Thus, in order to compensate for changes in excitability induced by GABA_A_ activity and the associated reduction in membrane resistance, E_GABA_ must be higher than the threshold for action potential generation. In the case of no change in membrane resistance associated with GABA_A_ conductance, as is the case in the dynamic-clamp experiments, E_GABA_ appeared lower than the threshold for action potential. Since GABA_A_ currents tune excitability and spike timing, the reversal potential where GABA_A_ activity has the minimal impact appears to be the potential at which the GABA_A_ currents are on average equal to zero. Thus, the electrophysiological characteristics of a given neuron may be associated with a specific E_GABA_ such that the changes in excitability and reduction in spike timing reliability by GABAergic activity may be alleviated.

### Spike-timing and network activity

Neuronal coding of sensory information relies on the mean rate of action potential discharge but also the relative timing between action potentials. Stimulus-dependent changes in spike synchronization have been observed in various cortical areas, and it has been suggested that synchronization of spikes within a precise millisecond range could serve as an efficient mechanism to group neurons into cell assemblies responding to specific stimuli [54], [55]. During stimulus processing the recruitment of excitatory neurons and their connections are often the primary requisites for neuronal computation. GABAergic neurones contribute to synchronization. In invertebrates, GABA_A_ receptors were shown to be of critical importance as their blockade abolished oscillatory synchronization but not the individual responsiveness of projection neurones [56]. The presence of feed-forward inhibition or autaptic synapses in various areas of the central nervous system may be critical to maintain spike timing and synchrony [19], [20], [53]. Nevertheless, most of the phasic GABAergic activity appears not to be correlated with sensory stimuli [26], tonic inhibition is present in different brain areas [21] and asynchronous release of GABA is observed in CCK-interneurones in the hippocampus [57]–[59]. Thus, synaptic and extra-synaptic GABA_A_ receptors can be activated in various modes, which share common characteristics but also particular features that may have computational significance for the regulation of network activity.

In conclusion, I have emphasized in this study the fact that, in addition to the well-documented modulation of neuronal excitability, spontaneous GABAergic activity has a major detrimental impact on spike timing reliability. A future challenge is to understand how variability in spike timing contributes to neuronal processing in the brain, in this context the impact of GABA release and chloride homeostasis in deteriorating spike timing should not be neglected.

## Materials and Methods

### Ethics Statement

All rats were maintained on a 12 h light/dark cycle with food and water provided ad libitum. The research involving animals has been approved by the Direction Départementale des Services Vétérinaires – Préfecture des Bouches du Rhône, France (permit number C13-055-8), and the Institut Jean-Roche Animal Care Supervisor (M. Mekahouche, D.V.M Ph.D, permit number 13-122). All experiments were carried out according to the European and Institutional guidelines for the care and use of laboratory animals (Council Directive 86/609/EEC and French National Research Council).

### Cortical slice preparation and electrophysiological recordings

Transverse cortical slices (350–400 µm thick) were obtained from 13- to 20-day old Wistar rats as previously described [60]. Experiments were performed at 32°C in ACSF containing the following (in mM): NaCl 125, NaHCO3 26, CaCl2 3, KCl 2.5, MgCl2 2, NaH2PO4 0.8, D-glucose 10, kynurenate (2 mM); and saturated with 95% O2 and 5% CO2. L2/3 pyramidal cells (recorded between 300 and 800 µm from the pia) were visualised using an Olympus BX-51 WI microscope equipped with differential interference contrast optics under infrared illumination and a water immersion lens (X60, 0.9 NA, Olympus). Unless otherwise stated, electrophysiological recordings were performed in whole-cell configuration with a Multiclamp 700B amplifier (Axon Instruments), filtered at 5 kHz and digitized at 20 kHz via a PCI-6220 National Instrument interface controlled by IgorPro (Wavemetrics) and/or Digidata 1322A interface controlled by PClamp software (Axon Instruments). Patch pipettes had a resistance of 3–6 MΩ when filled with a solution containing (in mM): K-gluconate 120, KCl 20, HEPES 10, EGTA 0.5, Na2ATP 2, NaGTP 0.3, and MgCl2 2, pH 7.4. Cells were recorded if the series resistance, measured throughout experiments, remained stable and <20 MOhm. In a subset of experiments (Fig. 1, Fig. S1c,d), perforated patch-clamp recordings were performed. Gramicidin (100 µg/ml) was added to the intracellular solution. For this recording configuration the tip of the pipette was backfilled with the solution containing no gramicidin to allow a good cell-attached configuration prior to perforation by gramicidin diffusion at the tip of the pipette. Alexa 488 or 568 (Molecular Probes) were added to the intracellular solution in order to confirm the pyramidal cell morphology in some whole-cell recordings. Fluorescence excitation was performed using a PolyV monochromator (Till photonics). Fluorescence imaging was performed with a CCD Camera (CoolSnap HQ2). Both excitation patterns and fluorescence acquisition were controlled by Metavue (Molecular Devices) and IgorPro (Wavemetrics) software. Isoguvacine (0.5 mM) was pressure ejected using a pico-Spritzer (TooheySpritzer). Typical pressure and time ejection were 7PSI and 30 ms respectively. Alexa 588 (Molecular Probes) was added to the isoguvacine solution in order to visualise its diffusion when ejected. During the course of the perforated patch-clamp recordings, E_GABA_ was estimated from the polarity of isoguvacine responses at different holding membrane potential. This protocol inform me, together with alexa 488 dye imaging that perforated patch did not evolve into whole cell mode during the course of the experiment, Electrophysiological recordings were stopped if a sudden change in E_GABA_ or fluorescence of the soma was observed.

Picrotoxin (100 mM) was prepared in ethanol and stored at −80°C. Kynurenate (200 mM) was prepared in distilled water and stored at −20°C (respectively). Stocks solutions were thawed and diluted into the extracellular solution before use.

### Stimuli

Voltage or DC steps, GABA_A_ synaptic conductances and reversal potentials were constructed, according to experimental needs with Igor Pro software (Wavemetrics), and converted to analog signals via a PCI-6723 National Instrument interface. GABA_A_ like currents were simulated with a dynamic-clamp amplifier (SM1, Cambridge Conductance), using for inputs the conductance profile, the reversal potential and the membrane potential of the recorded cell. The calculated current was summed to the DC step and injected into the recorded cell.

### Neuronal modelling

GABAergic synaptic events were modelled as a conductance with a dual exponential time course of the form (1-exp(-t/tau_rise_).exp(-t/tau_decay_). Spontaneous activity was created by convolution of the GABAergic event template with the Poisson train occurrence of spontaneous events. The conductance instruction was then either converted to an analog signal to instruct the dynamic clamp amplifier or injected to the computational model. The latter was based on a leaky integrate and fire model (LIF) with random threshold [23], [24] unless otherwise stated: Cm.dV_i_/dt = Sigma_i_.g_i_.(V_O_−V_i_)+Sigma_i_.I with C_m_ = 400 pF; V_o_ = −65 mV. When V_i_ reaches a threshold theta, a spike is generated and the membrane potential resets to V_o_. Resting membrane resistance was set at 200 MOhm. GABAergic synaptic events, affecting both g_i_ and I were sometimes included, according to the equation I_GABA_ = g_GABA_.(Vm−E_GABA_). E_GABA_ was set at −70 mV unless otherwise stated. All modelling was performed with Igor Pro Software.

### Analysis

Spontaneous synaptic activity was analyzed with the help of Minianalysis (Synaptosoft, Decatur, GA, USA). Spike analysis was performed with Igor Pro (Wavemetrics, Lake Oswego, OR, USA), Neuromatic functions (Jason Rothman, http://www.neuromatic.thinkrandom.com) were used in addition to homemade functions. 3D graphs were drawn under Origin Pro 8.0 (Origin Lab, Northampton, MA, USA) by smoothing average data with a thin plate spline function.

Spike Threshold was detected when dV/dt exceeded 10 mV/ms. Measurements of CV_ISI_ were performed by averaging CV for each sequential interval along the discharge starting from the 2^nd^ spike. With this method, the reproducibility of the full discharge for one trial to another could be evaluated, independently of spike-frequency adaptation that was observed experimentally.

Measurement of spike time jitter @ 10 spikes/s was performed by interpolating the CV_ISI_ *vs* average spike discharge with either the best linear or exponential fit.

Plots of firing probability (fig. 5) were obtained by convolving the times of spike generation with a Gaussian function with standard deviation equal to the duration of the sweep, divided by the mean number of spikes [61]. Statistical comparisons were made using ANOVA paired tests. Differences were considered significant when P<0.05. Data are reported as mean ± s.e.m.

## Supporting Information

Figure S1 **Characteristics of spontaneous GABA_A_ events received by L2/3 pyramidal cells.** a, Spontaneous occurrence of GABAergic current transients recorded in a L2/3 pyramidal cell (V_hold_ = −70 mV). Their frequency was on average 3.8±1.0 events/s (range 0.9–13.1 events/s, n = 15). b, Average GABA_A_ current transients (from n = 66 events) received by the cell shown in a. For all cells tested GABA_A_ current transients were displayed a fast 10–90% rise time (1.5±0.2 ms, range 0.6–3.7 ms, n = 15); the conductance at the peak of current was 343±28 nS (range 146–508 nS, n = 15); their tail was best fitted with a monoexponential decay (time constant 14.7±1.1 ms, range 7.2–21.4, n = 15). c, superimposed currents in response to a brief (15 ms) perisomatic pressure ejection of GABA_A_ agonist isoguvacine at 4 different V_hold_ (−88,−78,−68,−58 mv) during a gramicidin perforated voltage-clamp recording. d, GABA_A_ current measured at the peak of isoguvacine response *vs* V_hold_. Same cell as in c. E_GABA_ was on average −69±4 mV (range −82–−57 mV; n = 5).
(TIF)

Figure S2 **Leaky Integrate and Fire Model with Random Action Potential Threshold.** a, Electronic Design of the Leaky Integrate and Fire Model. It is based on an RC circuit with a condition: if Vm crosses the Threshold Θ, an action potential waveform lasting 10 ms, that peaks at +30 mV and resets at −60 mV, is added. b, Θ can be either invariable (green line) or random when adding a Gaussian noise to Θ (red SDV = 1 mV). c, Superimposed (5) Vm fluctuations of the LIF model in response to a DC step (1 s, 160 pA) and Waterfall view of the Vm in order to show the jitter of the previous and following spikes when the 5^th^ spike was set as a the time reference in control (green; SDV Θ = 0 mV), or when SDV Θ = 1 mV (red). d,. Mean firing rate *vs* DC step. Same colour code as in c. e, CV_ISI_ *vs* firing rate when SDV Θ = 1 mV. CV remains null if SDV Θ = 0 mV.
(TIF)

Figure S3 **Contributions of GABA_A_ current and GABA_A_ shunt to the frequency-dependent tuning in Excitability and Fidelity.** a, From upper to lower, a G_GABA-A_ pattern of 33 events/s, Vm, sum of the DC step (135 pA)+I_GABA-A_ and membrane input resistance (R_in_) fluctuations in the LIF model when E_GABA_ was set at −70 mV. b, Same conditions as in a but R_in_ remained constant despite transient changes in G_GABA-A_. c, Same conditions as in a but I_GABA-A_ remained null during the simulation despite transient changes in G_GABA-A_. d, Mean firing rate displayed on a pseudocolor scale *vs* DC step and the rate of randomly occurring GABA_A_ activity in control conditions (left), when GABA_A_ activity induces only transient changes in GABA_A_ currents (middle) and when GABA_A_ activity induces only transient changes in R_in_ (right). e, CV_ISI_ displayed on a pseudocolor scale *vs* firing and GABA_A_ activity rate. Same conditions as in d.
(TIF)

Figure S4 **Amplitude- and Frequency-dependent tuning of pyramidal cell discharge fidelity by randomly occurring GABA_A_ conductance transients.** a, From upper to lower, a G_GABA-A_ pattern of 33 events/s, Vm, sum of the DC step (135 pA)+I_GABA-A_ and membrane input resistance (R_in_) fluctuations in the LIF model when E_GABA_ was set at −70 mV. Peak GABA_A_ conductance was set at 0.5 nS. b, Same conditions as in a but peak GABA_A_ conductance was set at 1 nS. c, Same conditions as in a but peak GABA_A_ conductance was set at 2 nS. d, Mean firing rate displayed on a pseudocolor scale *vs* DC step and the rate of randomly occurring GABA_A_ activity when Peak GABA_A_ conductance was set at 0.5 nS (left), 1 nS (middle) and 2 nS (right). e, CV_ISI_ displayed on a pseudocolor scale *vs* firing and GABA_A_ activity rate. Same conditions as in d.
(TIF)

Figure S5 **The kinetics of randomly occurring normalised GABA_A_ conductance transients do not affect the frequency-dependent tuning of Excitability and Fidelity.** a, From upper to lower, a G_GABA-A_ pattern of 33 events/s, Vm, sum of the DC step (135 pA)+I_GABA-A_ and membrane input resistance (R_in_) fluctuations in the LIF model when E_GABA_ was set at −70 mV. Peak GABA_A_ conductance was set at 2.005 nS, and GABA_A_ conductance transients had fast kinetics (Tau_rise_ = 0.5 ms; Tau_decay_ = 5 ms). b, Same conditions and GABA_A_ charge as in a but GABA_A_ conductance transients had medium kinetics (Peak GABA_A_ conductance = 1 nS; Tau_rise_ = 1 ms; Tau_decay_ = 10 ms). c, Same conditions as in a but GABA_A_ conductance transients had slow kinetics (Peak GABA_A_ conductance = 0.2268 nS; Tau_rise_ = 10 ms; Tau_decay_ = 30 ms). d, Mean firing rate displayed on a pseudocolor scale *vs* DC step and the rate of randomly occurring GABA_A_ activity when GABA_A_ conductance transients had parameters depicted in a (fast kinetics, left), when GABA_A_ conductance transients had parameters depicted in b (medium kinetics, middle) and when GABA_A_ conductance transients had parameters depicted in c (slow kinetics, right). e, CV_ISI_ displayed on a pseudocolor scale *vs* firing and GABA_A_ activity rate. Same conditions as in d.
(TIF)
