# Supplementary figures and images for: Pre & Postsynaptic Tuning of Action Potential Timing by Spontaneous GABAergic Activity
Source: PLoS One. 2011 Jul 15;6(7):e22322. doi: 10.1371/journal.pone.0022322 (PMC3137631; doi:10.1371/journal.pone.0022322)

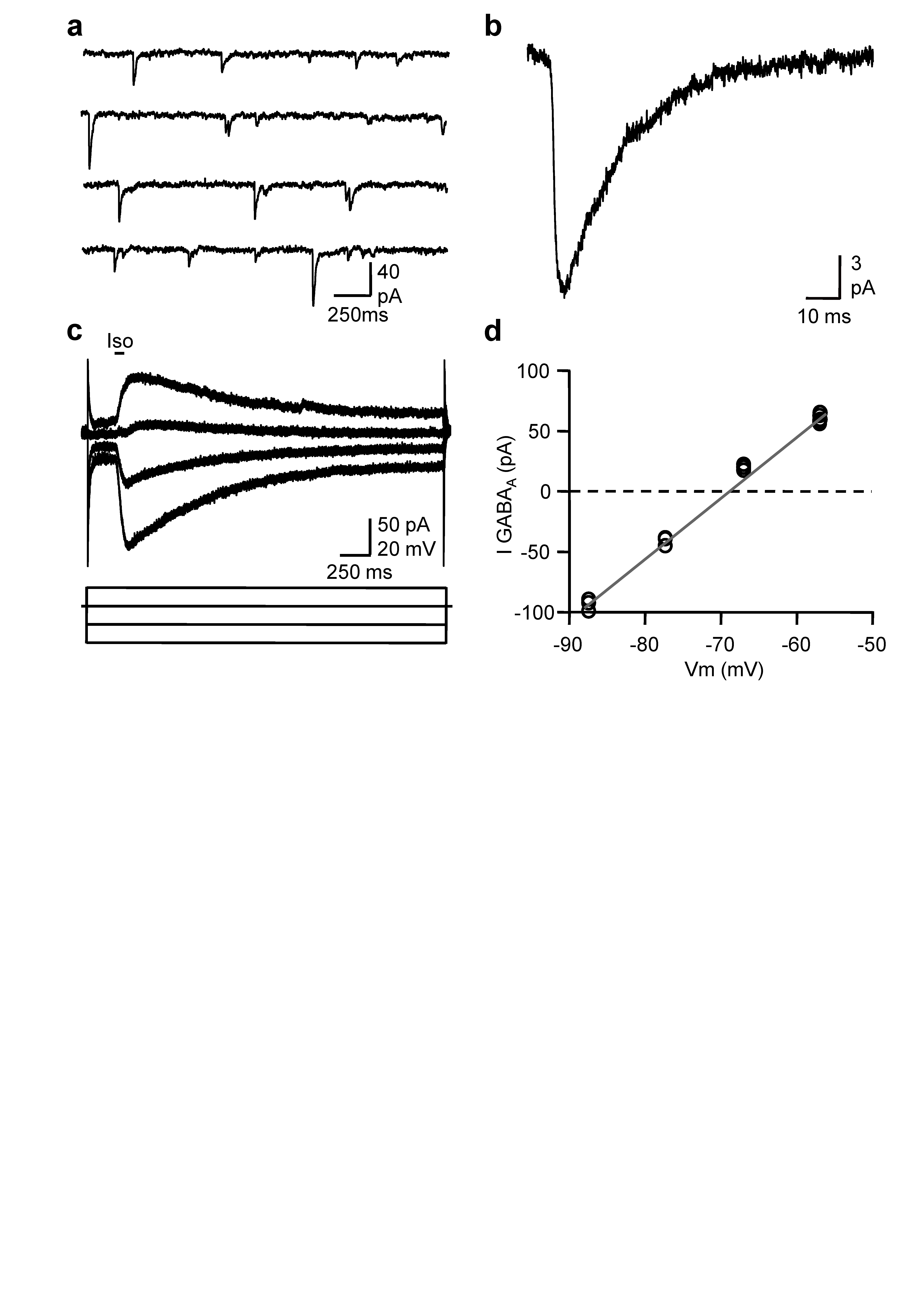

Supplement: Figure S1 — Characteristics of spontaneous GABAA events received by L2/3 pyramidal cells. a, Spontaneous occurrence of GABAergic current transients recorded in a L2/3 pyramidal cell (Vhold = −70 mV). Their frequency was on average 3.8±1.0 events/s (range 0.9–13.1 events/s, n = 15). b, Average GABAA current transients (from n = 66 events) received by the cell shown in a. For all cells tested GABAA current transients were displayed a fast 10–90% rise time (1.5±0.2 ms, range 0.6–3.7 ms, n = 15); the conductance at the peak of current was 343±28 nS (range 146–508 nS, n = 15); their tail was best fitted with a monoexponential decay (time constant 14.7±1.1 ms, range 7.2–21.4, n = 15). c, superimposed currents in response to a brief (15 ms) perisomatic pressure ejection of GABAA agonist isoguvacine at 4 different Vhold (−88,−78,−68,−58 mv) during a gramicidin perforated voltage-clamp recording. d, GABAA current measured at the peak of isoguvacine response vs Vhold. Same cell as in c. EGABA was on average −69±4 mV (range −82–−57 mV; n = 5). (TIF) [file pone.0022322.s001.tif]

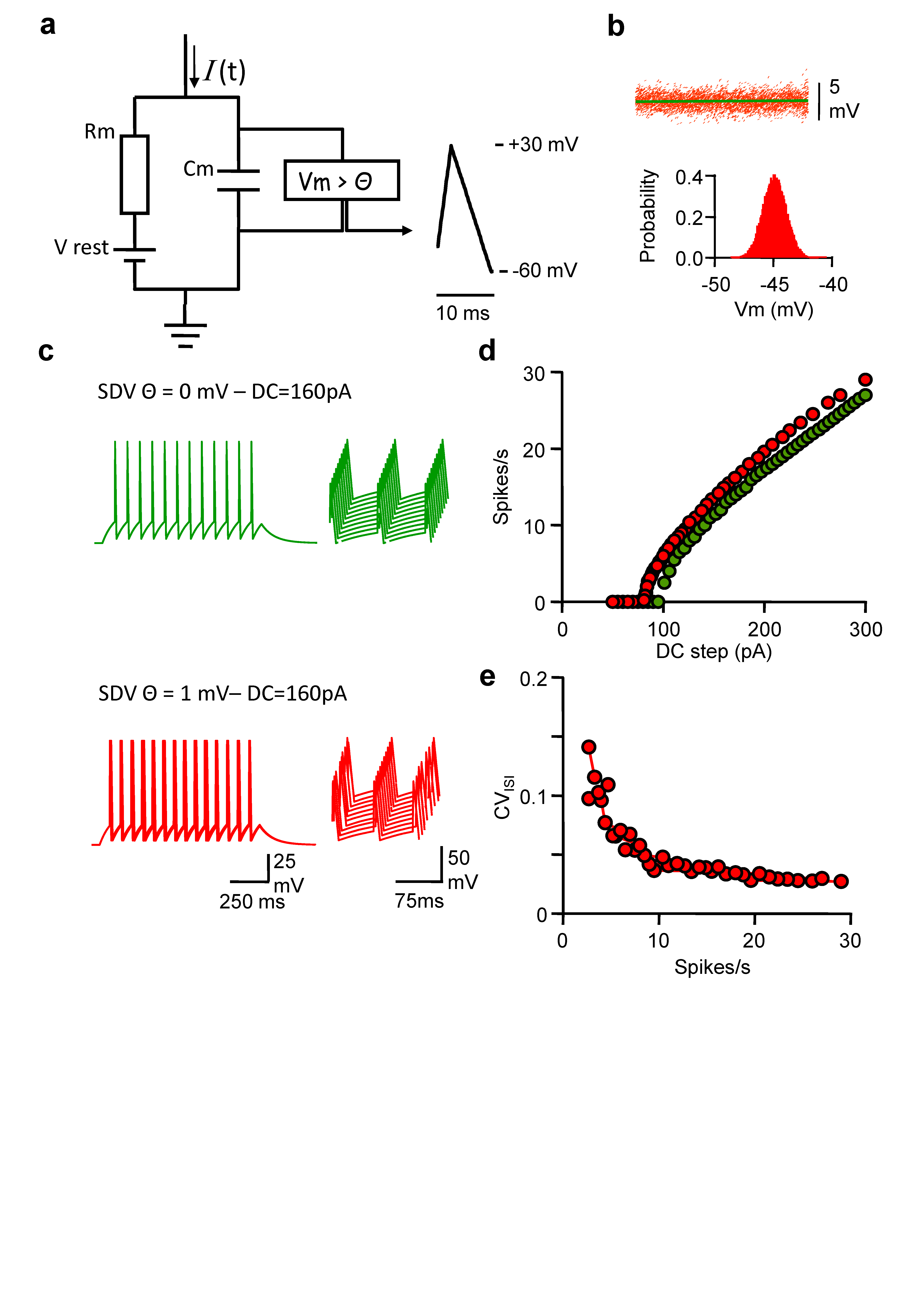

Supplement: Figure S2 — Leaky Integrate and Fire Model with Random Action Potential Threshold. a, Electronic Design of the Leaky Integrate and Fire Model. It is based on an RC circuit with a condition: if Vm crosses the Threshold Θ, an action potential waveform lasting 10 ms, that peaks at +30 mV and resets at −60 mV, is added. b, Θ can be either invariable (green line) or random when adding a Gaussian noise to Θ (red SDV = 1 mV). c, Superimposed (5) Vm fluctuations of the LIF model in response to a DC step (1 s, 160 pA) and Waterfall view of the Vm in order to show the jitter of the previous and following spikes when the 5th spike was set as a the time reference in control (green; SDV Θ = 0 mV), or when SDV Θ = 1 mV (red). d,. Mean firing rate vs DC step. Same colour code as in c. e, CVISI vs firing rate when SDV Θ = 1 mV. CV remains null if SDV Θ = 0 mV. (TIF) [file pone.0022322.s002.tif]

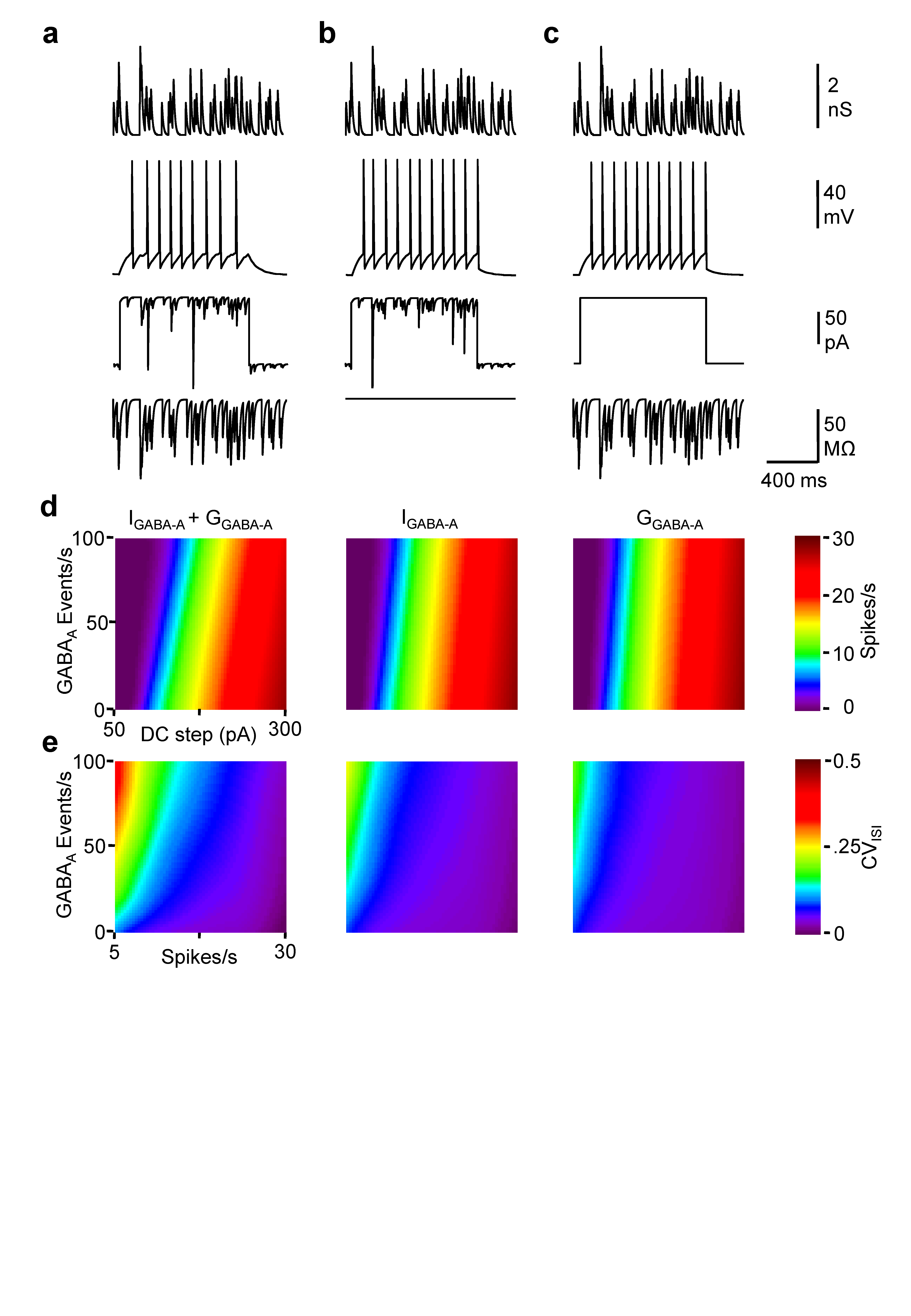

Supplement: Figure S3 — Contributions of GABAA current and GABAA shunt to the frequency-dependent tuning in Excitability and Fidelity. a, From upper to lower, a GGABA-A pattern of 33 events/s, Vm, sum of the DC step (135 pA)+IGABA-A and membrane input resistance (Rin) fluctuations in the LIF model when EGABA was set at −70 mV. b, Same conditions as in a but Rin remained constant despite transient changes in GGABA-A. c, Same conditions as in a but IGABA-A remained null during the simulation despite transient changes in GGABA-A. d, Mean firing rate displayed on a pseudocolor scale vs DC step and the rate of randomly occurring GABAA activity in control conditions (left), when GABAA activity induces only transient changes in GABAA currents (middle) and when GABAA activity induces only transient changes in Rin (right). e, CVISI displayed on a pseudocolor scale vs firing and GABAA activity rate. Same conditions as in d. (TIF) [file pone.0022322.s003.tif]

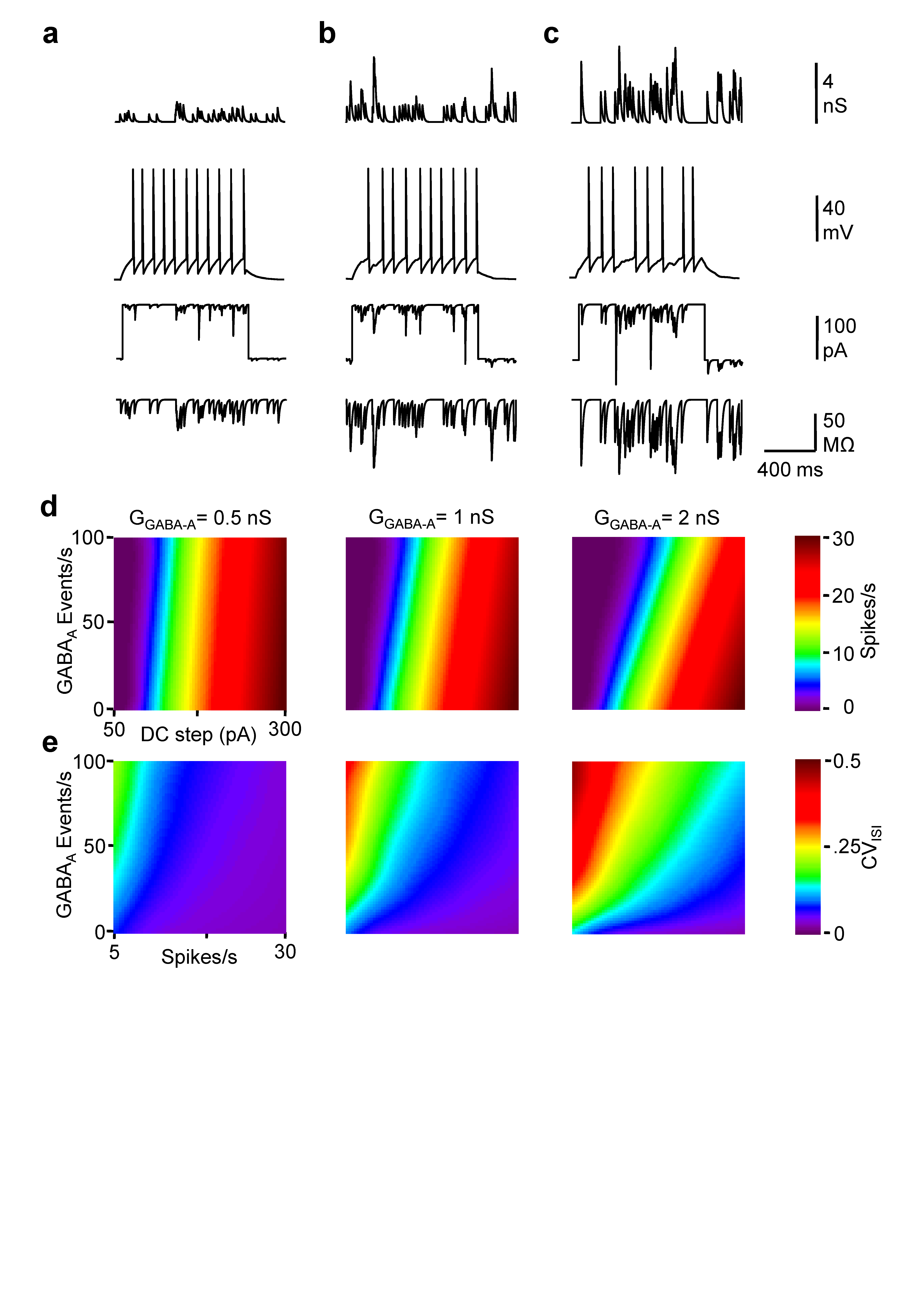

Supplement: Figure S4 — Amplitude- and Frequency-dependent tuning of pyramidal cell discharge fidelity by randomly occurring GABAA conductance transients. a, From upper to lower, a GGABA-A pattern of 33 events/s, Vm, sum of the DC step (135 pA)+IGABA-A and membrane input resistance (Rin) fluctuations in the LIF model when EGABA was set at −70 mV. Peak GABAA conductance was set at 0.5 nS. b, Same conditions as in a but peak GABAA conductance was set at 1 nS. c, Same conditions as in a but peak GABAA conductance was set at 2 nS. d, Mean firing rate displayed on a pseudocolor scale vs DC step and the rate of randomly occurring GABAA activity when Peak GABAA conductance was set at 0.5 nS (left), 1 nS (middle) and 2 nS (right). e, CVISI displayed on a pseudocolor scale vs firing and GABAA activity rate. Same conditions as in d. (TIF) [file pone.0022322.s004.tif]

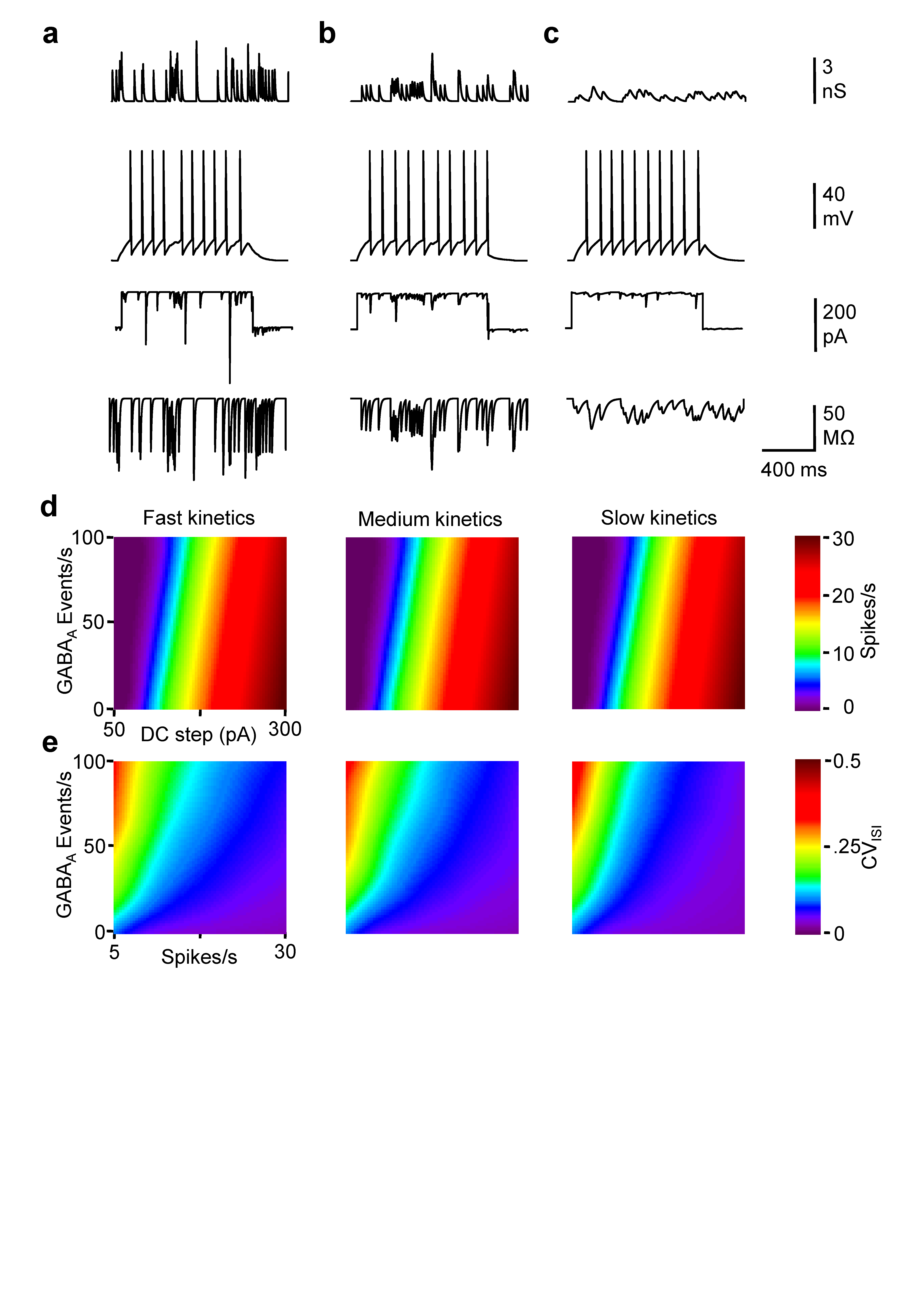

Supplement: Figure S5 — The kinetics of randomly occurring normalised GABAA conductance transients do not affect the frequency-dependent tuning of Excitability and Fidelity. a, From upper to lower, a GGABA-A pattern of 33 events/s, Vm, sum of the DC step (135 pA)+IGABA-A and membrane input resistance (Rin) fluctuations in the LIF model when EGABA was set at −70 mV. Peak GABAA conductance was set at 2.005 nS, and GABAA conductance transients had fast kinetics (Taurise = 0.5 ms; Taudecay = 5 ms). b, Same conditions and GABAA charge as in a but GABAA conductance transients had medium kinetics (Peak GABAA conductance = 1 nS; Taurise = 1 ms; Taudecay = 10 ms). c, Same conditions as in a but GABAA conductance transients had slow kinetics (Peak GABAA conductance = 0.2268 nS; Taurise = 10 ms; Taudecay = 30 ms). d, Mean firing rate displayed on a pseudocolor scale vs DC step and the rate of randomly occurring GABAA activity when GABAA conductance transients had parameters depicted in a (fast kinetics, left), when GABAA conductance transients had parameters depicted in b (medium kinetics, middle) and when GABAA conductance transients had parameters depicted in c (slow kinetics, right). e, CVISI displayed on a pseudocolor scale vs firing and GABAA activity rate. Same conditions as in d. (TIF) [file pone.0022322.s005.tif]
